# Cobicistat as a Potential Booster of Ponatinib and Dasatinib Exposure in a CML Patient: A Case Study

**DOI:** 10.1097/FTD.0000000000001103

**Published:** 2023-05-30

**Authors:** Susan Hofman, Daan J. Touw, Bart F. R. Span, Thijs H. Oude Munnink

**Affiliations:** * Department of Clinical Pharmacy and Pharmacology, University Medical Center Groningen, University of Groningen, the Netherlands; and; † Department of Hematology, University Medical Center Groningen, University of Groningen, the Netherlands.

**Keywords:** pharmacokinetic boosting, pharmacokinetic enhancement, cobicistat, tyrosine kinase inhibitors, chronic myeloid leukemia

## Abstract

The authors present a case of a 57-year-old patient with chronic myeloid leukemia who was treated with ponatinib and subsequently treated with dasatinib. The patient showed a major molecular response; however, the BCR-ABL1 signal increased with low ponatinib and dasatinib trough concentrations. Cobicistat was used as a pharmacokinetic booster to increase ponatinib and dasatinib exposure, as opposed to increasing the dose. However, ponatinib exposure was not sufficiently increased by cobicistat. The peak dasatinib concentration was successfully increased with cobicistat treatment. Dasatinib and cobicistat cotreatment induced a response in BCR-ABL1 PCR signal, was well tolerated, and led to a substantial reduction in drug costs.

## CLINICIAN

A 57-year-old patient with chronic myeloid leukemia and cytogenetic aberrations was treated with BCR-ABL1 tyrosine kinase inhibitors for 4 years. The patient was initially treated with imatinib (400 mg, once daily). As the 12-month response, milestone was not achieved and there was no cytogenetic remission; imatinib was switched to dasatinib (140 mg, once daily). BCR-ABL mutations were not detected. After 9 months of dasatinib treatment, the dasatinib trough serum concentration was low (<1 mcg/L) and there was inadequate response (PCR BCR-ABL, 0.16%). Therefore, treatment was switched to ponatinib (45 mg, once daily). Despite a low ponatinib serum concentration (8 mcg/L), major molecular response (MMR) was reached after 1 month. Based on this response, ponatinib treatment was continued at the same dose. After 18 months of treatment, the PCR signal increased from 0.0088% to 0.029%, while maintaining MMR. Adherence was discussed with the patient, and nonadherence was found to be of no concern because the patient was highly motivated to take medication at scheduled times. The serum ponatinib trough concentration was 12 mcg/L. Based on these findings, could there be a possibility of insufficient ponatinib exposure and is there an option to increase ponatinib exposure?

## TDM CONSULTANT

The population pharmacokinetic model predicted trough concentration of ponatinib at a dose of 45 mg once daily is 37.5 µL/L (with 90% prediction interval of 12.2–94.3 mcg/L).^1^ The ponatinib trough concentration of 12 mcg/L in this patient is at the lower range of the expected reference value. Exposure response or exposure toxicity–based target concentrations of ponatinib have not yet been established, but based on in vitro data, a serum concentration of >10.7 mcg/L is preferably maintained during the dosing interval for adequate BCR-ABL1 inhibition and optimal treatment response.^1,2^ Stating that the current trough concentration of 12 mcg/L is subtherapeutic is inaccurate. However, based on the relatively low trough concentration, good tolerability, and increase in the BCR-ABL1 PCR signal, an increase in the ponatinib dose may be considered to achieve higher exposure. As the current dose of 45 mg is the highest dose according to the manufacturer's label, a further increase in the dose would result in substantial financial implications. Another possibility is the use of a CYP3A4 inhibitor, such as cobicistat, as a pharmacokinetic booster for ponatinib exposure. Although ponatinib is only partially metabolized by CYP3A4, a drug–drug interaction study with the strong CYP3A4 inhibitor ketoconazole showed that ketoconazole increased the AUC_0-∞_, AUC_0-τ_, and C_max_ of ponatinib by 78%, 70%, and 47%, respectively.^3^ Using an azole to boost ponatinib may lead to unwanted effects such as resistant *Candida* strains, as well as side effects. Adding the strong CYP3A inhibitor cobicistat is, therefore, a better strategy than using an azole and is expected to increase ponatinib exposure by approximately 50%–100%. Boosting ponatinib exposure will result in reduced exposure to the major metabolite AP24600, although this is expected to be without consequences since AP24600 is pharmacologically inactive.^3^ Since the patient is not taking other relevant concomitant medications, adding cobicistat is considered to be safe in this patient. Because of the low ponatinib and dasatinib concentrations, it may also be of interest to perform CYP3A4 and CYP3A5 genotyping. Pharmacogenetic polymorphisms can influence the activity of enzymes involved in ponatinib and dasatinib metabolism, leading to reduced exposure.

## CLINICIAN

Cobicistat (150 mg once daily) was added to the treatment. After 1 week of cotreatment with cobicistat, the patient felt well. Ponatinib trough concentration increased to 17 mcg/L.

## TDM CONSULTANT

After 1 week of treatment, steady-state ponatinib concentrations could possibly not have been reached, since the elimination half-life of 22 hours might be extended by CYP3A4 inhibition.^3,4^ The ponatinib trough concentration was measured again in 1 week to check steady-state exposure.

## CLINICIAN

The patient felt well after 2 weeks of cobicistat cotreatment. The ponatinib trough concentration was 17 mcg/L. What are the next steps?

## TDM CONSULTANT

The steady-state concentration of cobicistat-boosted ponatinib was reached after 2 weeks of combined treatment. The increase in ponatinib trough concentration induced by cobicistat was only moderate, and the ponatinib trough concentration remained lower than the average trough concentration. Genotype analysis will require an additional week, so continue treatment at the current dosage.

## CLINICIAN

No pharmacogenetic polymorphisms for CYP3A4 and CYP3A5 were found in the genotype analysis; the CYP3A4 genotype was normal (CYP3A4*1/*1), and the patient was a CYP3A5 nonexpressor (CYP3A5*3/*3). The ponatinib trough concentration is now 14 mcg/L.

## TDM CONSULTANT

The ponatinib trough concentration remained lower than the average trough concentration observed at this dose. There was no evidence to suggest that the current concentration was subtherapeutic. A higher cobicistat dose of 150 mg (twice daily) has been used to boost axitinib and osimertinib,^5,6^ so an increase in the dose to 150 mg twice daily might increase CYP3A inhibition, thereby increasing ponatinib exposure.

## CLINICIAN

After a week of treatment with cobicistat 150 mg (twice daily), ponatinib trough concentration decreased to 13 mcg/L. An increase in cobicistat dose had no effect on ponatinib exposure. The BCR-ABL1 PCR (0.019%) was stable, and MMR remained constant.

## TDM CONSULTANT

Boosting ponatinib with cobicistat did not produce the desired effect. Switching to exposure-optimized dasatinib should be considered to improve the efficacy of chronic myeloid leukemia (CML) treatment. Previously, this patient had low dasatinib concentrations; however, dasatinib might be more sensitive to cobicistat boosting, as CYP3A4 is the major metabolizing enzyme. Dasatinib metabolite concentrations will be reduced by CYP3A4 boosting, which is not of relevance since dasatinib metabolites do not substantially contribute to the effect of dasatinib.^7^ Start dasatinib 140 mg once daily (without cobicistat) and check dasatinib peak concentrations after a week of treatment.

## CLINICIAN

Ponatinib was switched to dasatinib, and cobicistat was stopped. After a week of treatment with dasatinib 140 mg once daily, the dasatinib peak concentration was 30 mcg/L.

## TDM CONSULTANT

The dasatinib peak concentration is below the target value of >50 mcg/L.^8^ We expected that a dose increase to 180 mg once daily would not increase the dasatinib peak concentration to a therapeutic concentration. Therefore, the addition of cobicistat is recommended to boost dasatinib exposure. Strong CYP3A4 inhibition with ketoconazole has shown to increase dasatinib C_max_, AUC_0-24_, and T1/2 by 256%, 384%, and 164%, respectively.^9^ Adding cobicistat, therefore, is expected to increase the dasatinib peak concentration by 200–300%. The label advised dasatinib dose modification when combined with strong CYP3A4 inhibitors is a reduction from 140 mg to 40 mg once daily.^7^ As we aim to increase the dasatinib peak concentration by approximately 100%, reduce the dasatinib dose to 70 mg once daily and start cobicistat 150 mg once daily.

## CLINICIAN

After 1 week of treatment with dasatinib 70 mg once daily combined with cobicistat, the dasatinib peak concentration was 262 mcg/L. The patient experienced flushes, facial edema, and diarrhea.

## TDM CONSULTANT

**FIGURE 1.**
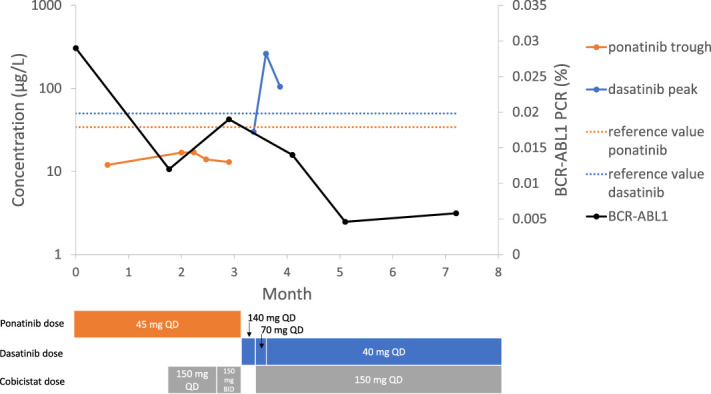
Ponatinib trough and dasatinib peak concentration before and during boosting with cobicistat. BCR-ABL1 PCR signal during treatment with ponatinib and dasatinib. Ponatinib reference value is the expected peak concentration of 37.5 mcg/L based on population pharmacokinetic modeling.^1^ Dasatinib reference value for peak concentration is a concentration of >50 mcg/L.^8^ BID, 2 times daily; QD, once daily.

The dasatinib peak concentration had increased by a factor 8.7 after adding cobicistat, while reducing the dasatinib dosage by 50%, as shown in Figure 1. This increase is greater than expected. The peak concentration is far above the target value of >50 mcg/L. Dasatinib toxicity has been related to the trough concentration, with more frequent pleural effusions in patients with trough concentration >3–4 mcg/L.^10^ Based on the high peak concentration and the toxicity the patient is experiencing, a dasatinib dose reduction from 70 mg to 40 mg once daily in combination with cobicistat is appropriate. Determine peak and trough concentrations after a week.

## CLINICIAN

After 1 week of treatment with dasatinib 40 mg once daily combined with cobicistat, the dasatinib peak concentration was 105 µm/L and trough concentration was 4 mcg/L. The patient was feeling well after administration.

## TDM CONSULTANT

The peak dasatinib concentration currently meets the target concentration. The trough concentration of 4 mcg/L is reasonably high, and the patient should be closely monitored for toxicity. As the patient is currently not experiencing toxicity, continue treatment and repeat trough concentration after 1 to 2 weeks.

## CLINICIAN

After 2 weeks of continued treatment with dasatinib 40 mg once daily combined with cobicistat, the dasatinib trough concentration was 4 mcg/L. The patient still showed MMR (BCR-ABL1, 0.014%) and was feeling well.

## TDM CONSULTANT

As the patient is currently not experiencing toxicity, the trough concentration is stable, the response is ongoing, and the treatment should be continued.

## CLINICIAN

After adequate dasatinib concentrations were achieved, the BCR-ABL1 PCR signal was decreased to 0.0058% after 4 months of treatment (Fig. 1). In another CML patient with an increasing BCR-ABL1 PCR signal and low dasatinib peak concentrations, a complete molecular response and adequate dasatinib concentrations were achieved after 2 months of treatment with cobicistat-boosted dasatinib (cobicistat 150 mg once daily and 20 mg once daily).

## CONCLUSIONS

Cobicistat can boost dasatinib exposure in patients with inadequate drug concentrations at high doses. Cobicistat-boosted exposure led to a decrease in BCR-ABL1 PCR signal and ongoing MMR. The use of cobicistat as a pharmacokinetic booster reduced dasatinib costs and was well tolerated. Boosting ponatinib treatment with cobicistat was ineffective in the present case.
